# Semantic Relationships Between Representational Gestures and Their Lexical Affiliates Are Evaluated Similarly for Speech and Text

**DOI:** 10.3389/fpsyg.2020.575991

**Published:** 2020-10-22

**Authors:** Sarah S. Hughes-Berheim, Laura M. Morett, Raymond Bulger

**Affiliations:** Department of Educational Studies in Psychology, Research Methodology and Counseling, University of Alabama, Tuscaloosa, AL, United States

**Keywords:** representational gesture, gesture comprehension, gesture-text relationship, gesture-speech relationship, Integrated Systems Hypothesis

## Abstract

This research examined whether the semantic relationships between representational gestures and their lexical affiliates are evaluated similarly when lexical affiliates are conveyed via speech and text. In two studies, adult native English speakers rated the similarity of the meanings of representational gesture-word pairs presented via speech and text. Gesture-word pairs in each modality consisted of gestures and words matching in meaning (semantically-congruent pairs) as well as gestures and words mismatching in meaning (semantically-incongruent pairs). The results revealed that ratings differed by semantic congruency but not language modality. These findings provide the first evidence that semantic relationships between representational gestures and their lexical affiliates are evaluated similarly regardless of language modality. Moreover, this research provides an open normed database of semantically-congruent and semantically-incongruent gesture-word pairs in both text and speech that will be useful for future research investigating gesture-language integration.

## Introduction

Gesture can be defined as hand or body movements that convey information (Özyürek, 2002; Melinger and Levelt, 2005). Most gestures are gesticulations (hereafter referred to simply as “gestures”), which are naturally produced in conjunction with speech (see Hostetter, 2011, for a review). According to McNeill, 1992, 2005 gesture taxonomy, deictic gestures indicate presence (or absence) of objects via pointing; beat gestures convey speech prosody and emphasis; and representational (i.e., metaphoric and iconic) gestures convey meaning relevant to co-occurring speech via form and motion. Representational gestures may be used to describe actions (e.g., swinging a bat), to depict spatial properties (e.g., describing a ring as round), or to refer to concrete entities associated with abstract ideas (e.g., putting a hand over one’s heart to convey love; Hostetter, 2011). Gesturing while speaking is so pervasive that gesture and speech have been argued to be inextricably integrated into mental representations of language (Kendon, 2000). The process of producing speech and gesture is thought to occur bi-directionally, such that speech production influences gesture production, and conversely, gesture production influences speech production (Kita and Özyürek, 2003).

By the same logic, gesture and speech are similarly integrated during language comprehension. The Integrated Systems Hypothesis (Kelly et al., 2010) posits that co-occurring gesture and speech interact bi-directionally during language processing to enhance comprehension. This interaction occurs obligatorily, such that information from one modality (speech) cannot be processed without being influenced by information from the other modality (gesture). This hypothesis is supported by behavioral findings indicating fast and accurate identification of an action in a prime video followed by a target video displaying semantically-congruent representational gesture and speech related to the prime. In contrast, identification of action in a prime video is relatively slow and inaccurate when it is followed by a target video containing gesture, speech, or both that are semantically-incongruent and partially unrelated to the prime. Further, even if instructions are issued to attend to speech and ignore accompanying gesture, error rates are higher when prime and target videos are semantically-incongruent than when they are semantically-congruent (Kelly et al., 2010).

The bi-directional and obligatory integration of gesture and speech postulated by the Integrated Systems Hypothesis has important implications for learning. Comprehension accuracy and speed are bolstered by viewing semantically-congruent representational gestures accompanying speech (Drijvers and Özyürek, 2017, 2020). Moreover, words learned with semantically-congruent representational gestures are remembered more accurately than words learned without gestures (Kelly et al., 2009; So et al., 2012). In addition to supporting the Integrated Systems Hypothesis, these findings are consistent with Dual Coding Theory (Clark and Paivio, 1991), which posits that representational gesture splits the cognitive load between the visual and verbal representational systems, freeing up cognitive resources and thereby enhancing comprehension. These findings suggest that when novel vocabulary is learned, it should ideally be accompanied by semantically-congruent representational gesture.

Importantly, not all representational gestures affect comprehension similarly. For example, representational gestures that are semantically-incongruent with lexical affiliates (i.e., associated words or phrases) disrupt comprehension even more than the absence of gesture (Kelly et al., 2015; Dargue and Sweller, 2018). Moreover, representational gestures frequently produced in conjunction with lexical affiliates (e.g., holding up one finger to simulate *first place*) benefit comprehension more than representational gestures infrequently produced in conjunction with the same lexical affiliates (e.g., outlining a ribbon with ones hands to simulate *first place*; Dargue and Sweller, 2018). Although both gestures convey the concept of *first place*, frequently-produced representational gestures are thought to enhance comprehension because such gestures are more semantically-related to co-occuring speech—and are therefore more easily processed—than infrequently-produced representational gestures (Woodall and Folger, 1981). By examining differences in language processing resulting from representational gestures that are related to co-occuring speech to varying degrees, these findings emphasize the importance of semantic congruency between gesture and speech in lightening cognitive load and thereby enhancing language comprehension.

Although extant research has examined the semantic relationship between representational gesture and speech, it is currently unknown whether the learning implications of the Integrated Systems Hypothesis (i.e., increased comprehension) extend to the semantic relationship between representational gesture and text. Similar to how speech conveys information acoustically, text conveys information orthographically and is therefore a component of mental representations of language (Özyürek, 2002; Melinger and Levelt, 2005). Unlike speech, however, text is comprehended within the visual modality; therefore, it must be processed sequentially with gesture. To our knowledge, no published research to date has investigated how the semantic relationship between representational gesture and text is represented, despite that text is the orthographic equivalent of speech.

Understanding whether gesture and text are integrated similarly to gesture and speech is crucial in furthering the understanding of gesture’s impact on language learning. When novel vocabulary is learned in instructional settings, words are often displayed in orthographic, as well as spoken, form. For example, a student may see a vocabulary word displayed on the white board or screen before seeing a gesture depicting what that word means. In order to determine whether representational gesture affects text comprehension in a similar manner to speech comprehension, it is first necessary to understand whether the semantic congruency of words presented via text with representational gestures is represented similarly to the semantic congruency of words presented via speech with representational gestures. Thus, the primary purpose of the present research was to compare how semantic congruency is represented, as evidenced by ratings, when representational gesture occurs with text vs. speech.

A secondary purpose of the present research was to provide an open normed database of semantically-congruent and semantically-incongruent gesture-word pairs in both text and speech for use in future research. Although a number of previous experiments have manipulated the semantic congruency of representational gestures and words relative to one another (Kelly et al., 2004, 2009, 2015; Özyürek et al., 2007; Straube et al., 2009; Dargue and Sweller, 2018), in most cases, the semantic congruency of gesture-word pairs was not normed. In light of this lack of norming data and evidence that the semantic relationship between representational gesture and lexical affiliates may fall along a continuum (Kelly et al., 2010; Dargue and Sweller, 2018), the degree of item-level variation within semantic congruence categories should be taken into consideration in future research. To minimize within-category variation in the present research, we constructed semantically-congruent gesture-word pairs from representational gestures and lexical affiliates (words) that they were consistently associated with, and we constructed semantically-incongruent gesture-word pairs from representational gestures and lexical affiliates with dissimilar, non-confusable forms and meanings.

To achieve our research objectives, we collected and compared semantic similarity ratings for representational gestures paired with semantically-congruent and semantically-incongruent words as speech and text. We predicted that semantically-congruent gesture-word pairs would be evaluated as highly semantically-related regardless of whether words were presented via text or speech, and that semantically-incongruent gesture-word pairs would be evaluated as highly semantically-unrelated regardless of whether words were presented via text or speech. These results would provide evidence that the semantic relationship between representational gesture and text, as evidenced by semantic congruency ratings, is represented similarly to the semantic relationship between representational gesture and speech.

## Method

### Participants

Two studies—a gesture-text and a gesture-speech study—were conducted via the internet with separate groups of participants. Sixty-nine participants were recruited for the gesture-text study, and seventy-one participants were recruited for the gesture-speech study. Participants (*n* = 140) were recruited from a large public university in the Southeastern United States in return for partial course credit. All participants were 18–35-year-old native English speakers who reported normal hearing and normal or corrected-to-normal vision and no speech, language, or learning impairments^1^. All participants provided consent to participate, and all experimental procedures were approved by the university’s institutional review board.

### Materials

All of the materials used in this research are publicly available via the Open Science Framework and can be accessed via the following link: https://osf.io/z5s3d/. Ninety-six English action verbs and 96 videos of representational gestures depicting their meanings (see Supplementary Appendix A) were used in the gesture-text and gesture-speech studies. Verbs were selected considering their frequency of use and the degree to which their meanings could be transparently conveyed via representational gesture. Representational gesture videos featured a woman silently enacting word meanings using the hands, body, and facial expressions. To ensure that spoken words did not differ in qualities such as affect, speed, or pitch based on their meanings, they were generated using the Microsoft Zira Desktop (Balabolka) text-to-speech synthesizer [English (United States, Female)].

Using these representational gesture and word stimuli, two types of gesture-word pairs were constructed for use in this study: Pairs consisting of gestures and words matched in meaning (semantically-congruent pairs), and pairs consisting of gestures and words mismatched in meaning (semantically-incongruent pairs; see Supplementary Appendix B). Construction of semantically-congruent and semantically-incongruent gesture-word pairs was based on data collected from a norming study in which 32 additional participants, who did not participate in the gesture-text or gesture-speech studies, selected the word best representing the action portrayed in each gesture video from among four alternatives. Based on this norming data, congruent gesture-word pairs were constructed from gestures reliably associated with their corresponding words, and incongruent gesture-word pairs were constructed from gestures and words with dissimilar, non-confusable forms and meanings.

Based on these gesture-word pairs, two lists were created for use in the gesture-text and gesture-speech studies. In these lists, gesture-word pairs were randomly divided in half and assigned to each congruency condition, such that gesture-word pairs that were semantically-congruent in one list were semantically-incongruent in the other list and vice versa. Order of presentation was randomized per participant such that semantically-congruent and semantically-incongruent gesture-word pairs were randomly interleaved in each study.

### Procedure

Participants were provided with an anonymized link to either the gesture-text or gesture-speech study. Upon following this link to initiate their respective studies, which were administered using the Qualtrics platform, participants were randomly assigned to one of the two lists of gesture-word pairs divided by semantic congruency described above.

**FIGURE 1:**
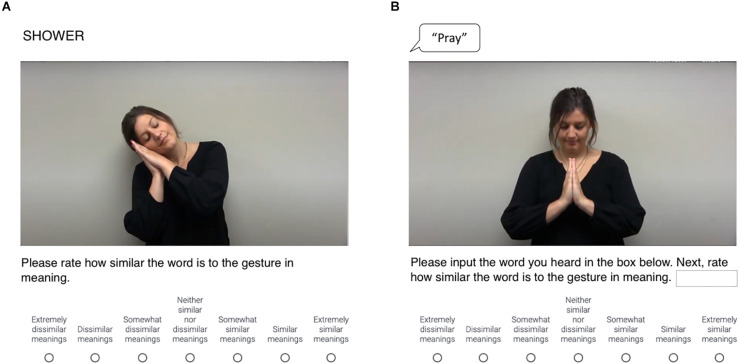
Schematic of **(A)** item featuring semantically-incongruent gesture-word pair from gesture-text task (Sleep—Shower); **(B)** item featuring semantically-congruent gesture-word pair from gesture-speech task (Pray—Pray).

In the gesture-text study, participants viewed words as text and subsequently watched video clips of representational gestures that were either semantically-congruent or semantically-incongruent with them. Participants then rated the similarity of the meanings of these words and gestures using a 7-point Likert scale ranging from 1 (extremely dissimilar) to 7 (extremely similar; see Figure 1A). In the gesture-speech study, participants played audio clips of spoken words and subsequently played video clips of representational gestures that were either semantically-congruent or semantically-incongruent with them. For each item, participants then typed the spoken word that they heard into a text box to ensure that they understood it correctly and subsequently rated the semantic similarity of the meaning of that word and the gesture that they had viewed using the same scale as the gesture-text study (see Figure 1B).

## Results

All of the data and analysis scripts used in this research are publicly available via the Open Science Framework and can be accessed via the following link: https://osf.io/z5s3d/. Table 1 displays frequency counts of semantic relatedness ratings by language modality and semantic congruency. Prior to analysis, words typed incorrectly in the gesture-speech study (22% of observations) were excluded. Semantic relatedness ratings for gesture-word pairs were then analyzed using a linear mixed effects model that included fixed effects of language modality and semantic congruency as well as random effects of participant and item with random slopes of congruency by participant, as follows:

lmer(rating∼modality×congruency)+(1+congruency|participant)+(1|item)

**TABLE 1:** Frequency of semantic relatedness ratings for gesture-word pairs by language modality and semantic congruency.

		Rating						
Language modality	Semantic congruency	Extremely dissimilar	Dissimilar	Somewhat dissimilar	Neither similar nor dissimilar	Somewhat similar	Similar	Extremely similar
		
		1	2	3	4	5	6	7
Speech	Congruent	13	24	39	52	351	711	1181
	Incongruent	1139	453	103	101	137	60	22
Text	Congruent	41	60	93	93	349	794	1161
	Incongruent	1295	640	180	121	211	105	39

This model was fit with Laplace estimation using the lmer() function of the lme4 package (Bates et al., 2015) in the R statistical programming language. Weighted mean-centered (Helmert) contrast coding was applied to all fixed effects (modality: speech = −0.54, text: 0.46; congruency: congruent = −0.48, incongruent: 0.52) using the psycholing package (Fraundorf, 2017) to obtain estimates analogous to those that would be obtained via ANOVA.

**FIGURE 2:**
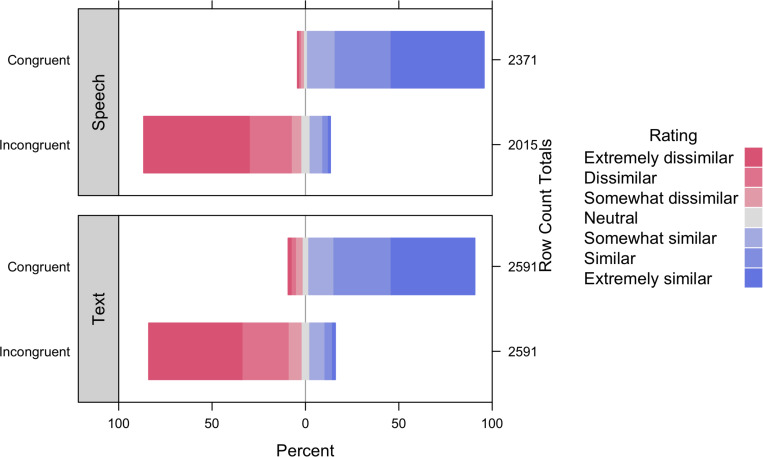
Percentage of semantic relatedness ratings assigned to gesture-word pairs by semantic congruency and language modality.

Table 2 displays parameter estimates for the model, and Figure 2 displays semantic relatedness ratings assigned to gesture-word pairs by semantic congruency and language modality. We observed a significant main effect of semantic congruency, indicating that the meanings of gestures and words in semantically-congruent pairs (*M* = 6.07; SD = 1.23) were rated as more similar than the meanings of gestures and words in semantically-incongruent pairs (*M* = 2.07; SD = 1.54). By contrast, the main effect of language modality failed to reach significance, indicating that the meanings of paired gestures and words were rated similarly regardless of whether words were presented via speech (*M* = 4.25; SD = 2.46) or text (*M* = 4.05; SD = 2.41). Although we observed a non-significant trend toward an interaction between semantic congruency and language modality, simple main effect analyses by language modality revealed that the meanings of gestures and words in semantically-congruent pairs were rated as more similar than the meanings of gestures and words in semantically-incongruent pairs both when words were presented via speech (*M*_congruent_ = 6.19, SD_congruent_ = 1.07; *M*_incongruent_ = 1.96, SD_incongruent_ = 1.48; *B* = −4.27, SE = 0.12, *t* = −35.21, and *p* < 0.001), as well as text (*M*_congruent_ = 5.96, SD_congruent_ = 1.35; *M*_incongruent_ = 2.14, SD_incongruent_ = 1.58; *B* = −3.81, SE = 0.11, *t* = −35.42, and *p* < 0.001). Likewise, simple main effect analyses by semantic congruency revealed that the meanings of gestures and words presented via speech and text were rated similarly regardless of whether their meanings were congruent (*B* = 0.08, SE = 0.09, *t* = 0.95, and *p* = 0.34) or incongruent (*B* = −0.08, *SE* = 0.10, *t* = −0.80, and *p* = 0.43). Together, these findings indicate that the semantic relatedness of gesture-word pairs is not affected by language modality (speech vs. text).

**TABLE 2:** Fixed effect estimates (Top) and variance estimates (Bottom) for multi-level linear model of semantic relatedness ratings of gesture-word pairs (observations = 9568).

Fixed effect	Coefficient	*SE*	Wald *z*	*p*
Intercept	4.14	00.07	62.89	< 0.001***
Semantic congruency	–3.86	0.17	–22.44	< 0.001***
Language modality	–0.03	0.06	–0.60	0.55
Semantic congruency × language modality	–0.25	0.14	–1.80	0.07^†^

**Random effect**				***s*^2^**

Participant				0.34
Participant × semantic congruency				1.28
Item				0.75

^†^*p* < 0.1; ^***^p < 0.001.

## Discussion

The present research investigated how semantic congruency is evaluated when representational gesture is paired with lexical affiliates (words) conveyed via text vs. speech. Consistent with our hypothesis that semantic congruency ratings for semantically-congruent and semantically-incongruent gesture-word pairs would not differ based on whether words were presented via text or speech, the results indicate that ratings differed by semantic congruency but not language modality. These findings provide evidence that the semantic relationship between representational gestures and their lexical affiliates is evaluated similarly regardless of whether lexical affiliates are conveyed via the spoken or written modality.

These preliminary findings can be leveraged to further investigate whether semantically-congruent representational gesture is as beneficial to text comprehension as it is to speech comprehension, as posited by the Integrated Systems Hypothesis. The Integrated Systems Hypothesis postulates that representational gesture and speech are obligatorily and bi-directionally processed to enhance language comprehension (Kelly et al., 2010). Although integration was not directly investigated in the current study using online measures, similar congruency ratings for gesture-word pairs presented via speech and text indicate that the semantic relationships between representational gestures and their lexical affiliates are evaluated similarly during both speech and text processing. Therefore, we hypothesize that representational gesture may be obligatorily and bi-directionally integrated with text, similar to speech, during language processing.

Future research should further probe the relationship between gesture-speech and gesture-text processing using additional methods. Online behavioral measures such as reaction time, eye-tracking, and mouse-tracking may provide further evidence of whether representational gesture is integrated with text similarly to how it is integrated with speech during language processing. Moreover, building on previous evidence that gesture and speech are processed simultaneously during language comprehension (Özyürek et al., 2007), cognitive neuroscience methods with high temporal resolution, such as event-related potentials, can illuminate whether gesture and text are integrated simultaneously during language comprehension, similar to gesture and speech. Finally, cognitive neuroscience methods with high spatial resolution, such as functional magnetic resonance imaging, can be leveraged to further investigate the extent to which functional activity subserving gesture-text integration overlaps with functional activity subserving gesture-speech integration (Willems et al., 2007).

Although cognitive load was not assessed directly in the current research, the results provide preliminary evidence supporting further investigation into whether semantically-congruent representational gesture accompanied by speech and semantically-congruent representational gesture accompanied by text reduces cognitive load, benefiting language comprehension (Kelly et al., 2009, 2010). Cognitive Load Theory indicates that splitting cognitive resources between the verbal and visuospatial representational systems may decrease the cognitive demands of language processing, thereby improving comprehension (Clark and Paivio, 1991). Future research should directly investigate the effect of semantically-congruent representational gesture on cognitive load during text comprehension by measuring comprehenders’ cognitive load while processing text accompanied by semantically-congruent representational gesture. Based on the findings of the current research, we hypothesize that representational gesture semantically related to sequentially-occurring language in both the spoken and written modalities may split comprehenders’ cognitive load between the verbal and visuospatial representational systems, thereby enriching representations of language during comprehension.

In addition to providing insight into the similarity of semantic congruency between representational gesture and text vs. representational gesture and speech, the present research provides an open database of semantically-congruent and semantically-incongruent gesture-word pairs normed for semantic relatedness in both text and speech. These stimuli and ratings will be useful for future research investigating how semantic congruency of representational gesture affects processing of spoken vs. read language, particularly with respect to controlling for item-level variability within semantic congruence categories. Thus, we hope that future research will utilize these materials to further illuminate the cognitive and neural mechanisms of gesture-language integration.

In sum, the results of the present research indicate that the semantic relationship between representational gesture and text is evaluated similarly to the semantic relationship between representational gesture and speech. Thus, these results provide preliminary evidence for future research to examine whether language processing—and learning and memory—may be enhanced not only by semantically-congruent representational gesture occurring with speech, but also with text. In particular, these results provide preliminary evidence in support of an investigation into whether language, regardless of the modality it is presented in (i.e., spoken or orthographic), is influenced by the semantic congruency of representational gesture, providing important insight into how the relationship between gesture and language is represented in the minds of comprehenders. The results of the current work may have important educational implications as vocabulary words are sometimes orthographically displayed and accompanied by representational gestures. Finally, this work provides an open source database of stimuli and ratings that can be used to investigate how semantically-congruent and semantically-incongruent representational gestures affect spoken and written language comprehension.

## Data Availability Statement

The datasets presented in this study can be found in online repositories. The names of the repository/repositories and accession number(s) can be found below: Open Science Framework: https://osf.io/z5s3d/

## Ethics Statement

The studies involving human participants were reviewed and approved by University of Alabama Institutional Review Board. The patients/participants provided their written informed consent to participate in this study. Written informed consent was obtained from the individual(s) for the publication of any potentially identifiable images or data included in this article.

## Author Contributions

SSH-B developed gesture and word stimuli, oversaw implementation and data collection for norming and gesture-text studies, drafted and edited introduction and discussion, and created Figure 1. LMM conceptualized research, analyzed data, drafted results, edited entire manuscript, and created tables and Figure 2. RB oversaw implementation and data collection for gesture-speech study, drafted and edited methods, and created Supplementary Appendices A,B. All authors contributed to the article and approved the submitted version.

## Conflict of Interest

The authors declare that the research was conducted in the absence of any commercial or financial relationships that could be construed as a potential conflict of interest.

## Footnotes

1 These criteria were used for pre-screening. No demographic data were collected.
