# Supplementary material for: Semantic Relationships Between Representational Gestures and Their Lexical Affiliates Are Evaluated Similarly for Speech and Text
Source: Front Psychol. 2020 Oct 22;11:575991. doi: 10.3389/fpsyg.2020.575991 (PMC7642993; doi:10.3389/fpsyg.2020.575991)
Supplement: Supplementary file 1 [file Table_1.docx]

**Appendix A**

Lexical Items and Semantically-Congruent Representational Gestures

| Lexical item | Semantically-congruent representational gesture |
| --- | --- |
| Add | The right and then the left hand, in vertically-oriented fists with fingers facing away from the body, are lifted vertically and are then moved towards each other horizontally until the knuckles touch. |
| Answer | The right hand, oriented horizontally with thumb and pinky extended and the remaining fingers curled against the palm, is brought to the right ear as the head is tilted slightly towards it. |
| Ask | The right hand, with the palm facing outwards and the fingers spread, is raised vertically above the head. |
| Bake | Right hand, oriented horizontally with palm facing outward and fingers and thumb extended and clasped, is moved forward away from body and retracted. Both hands, oriented horizontally with palms facing upwards and fingers and thumb extended together, are then brought forward separately and concurrently. |
| Blow | The right hand, open with fingers loosely curled and oriented with the knuckles outward, is positioned in front of an open mouth with the index finger and thumb touching the corners, as air is inhaled and then exhaled through the mouth. |
| Call | The left hand, with thumb and pinky extended and the remaining fingers curled against the palm, is oriented vertically with the fingers facing outwards, and the right hand, with index finger extended, is moved towards it and is placed near the curled fingers successively at different locations. The left hand is then moved beside the left ear as the head is tilted slightly towards it. |
| Catch | The right hand, with cupped palm facing outwards, is raised upwards, and the left hand is quickly brought across the body to clasp it. |
| Change | Both arms are crossed across the body at the waist as head is oriented to look downward. Arms are then concurrently brought above head, which is re-oriented to look forward, and then downward to either side. |
| Cheer | Both hands, in vertically-oriented fists with palms facing outward, are raised above the head on either side of the body and shaken horizontally several times while smiling. |
| Choke | Both hands, oriented vertically with the palms facing inwards and the fingers extended together and the thumbs oriented upwards, are concurrently brought to the neck as the eyes are opened widely. |
| Clap | Both hands, oriented vertically with the palms facing each other, are brought together horizontally until the palms touch three times. |
| Clean | The right hand, horizontally-oriented with fingers curled under and facing outward, is moved away from the body and back three times. |
| Click | The index finger of the right hand, oriented horizontally with palm facing downwards and fingers curved, is moved upward and then downward. |
| Climb | The right and left hands, with fingers apart and curled and palms facing outward, are placed in successively higher locations above one another alternately three times. |
| Cook | The right hand, oriented vertically with the thumb and fingertips clasped, is moved in several small horizontal circular motions, and then the left hand is positioned above it as the left hand’s loosely-curled fingers are moved in a sprinkling motion. |
| Cool down | The right hand is brought beside the face and is moved in a vertical fanning motion several times with fingers extended. |
| Cough | The right arm, with the elbow bent at a 90° angle, is extended horizontally to the right with a fisted hand, and the head is moved into the elbow as the mouth opens slightly and the head jerks forward twice. |
| Count | The right index, middle, and ring fingers are consecutively lifted upward into a vertical orientation from the fisted right hand. |
| Cross | The right hand is placed against the forehead, oriented horizontally with the palm downward, and the head is turned to look left and then right. The index and middle fingers are extended from a horizontally-oriented fisted right hand with the palm downwards and alternately moved forward as the hand is moved outward from the body. |
| Cry | Both hands, in horizontally-oriented fists with knuckles facing each other, are brought to the eyes and rotated inward towards each other twice as the eyes close and the bottom lip is extended. |
| Dance | The right hand, with index finger extended and remaining fingers curled in a horizontally-oriented fist, is moved across the body diagonally from left of the waist to right of the head twice while it is turned so that the index finger is pointing upward at the end of each motion. |
| Dig | The right hand, in an underhanded grip, and the left hand, in an overhanded grip, are separately and concurrently moved in a diagonal downward motion across the body until coming to a stop to the left of the hips. The hands are then turned and moved in a smaller, opposite diagonal upward motion until coming to a stop next to the right shoulder as horizontal fists with the knuckles parallel while turning the body sideways. |
| Dip | The right hand, with the thumb and fingertips clasped together, is moved in a U-like motion, ending with the hand oriented horizontally with fingers to the left. |
| Draw | The right hand, with index finger and thumb clasped together, is moved horizontally in wavy, looping motions. |
| Drink | The right hand, with fingers curled together, is brought to the mouth as the head is tilted back slightly. |
| Drive | Both hands, in vertically oriented fists with the thumbs on top, are concurrently extended and moved in a partial vertical circle, with the right hand moving upward and left and the left hand moving donward and right. This sequence is repeated in the opposite and then the same direction. |
| Dry off | The left arm is extended horizontally outward from the body while the right hand is brought along it from shoulder to the hand, and then the right arm is extended horizontally outward from the body while the left hand is brought along it from shoulder to the hand. |
| Eat | The right hand, with the knuckles bent at a 45° angle and the fingers extended inwards together, is moved vertically to the level of the mouth, which is opened and closed. |
| Erupt | Both hands, in a vertical orientation with fingers curled together and fingers oriented away from body, are brought together horizontally until the knuckles touch. Hands are then moved upward and apart in an arc-like motion as fingers are released into an extended position. |
| Flip | The right hand with the thumb and fingertips clasped and the palm oriented upwards is moved in a vertical circle, ending with the palm oriented downwards. |
| Fold | The thumb and fingertips of the right and left hand are clasped successively in a vertically-oriented position, and the wrists are then rotated towards the body in a circular motion, ending with clasped hands oriented away from the body with palms upward. |
| Go | Both hands, oriented horizontally away from the body with fingers extended together, are placed together, and then the right hand is moved horizontally away from the body while the left hand remains stationary. |
| Grade | The right hand, with the index finger and the thumb clasped together, is used to trace an “A” vertically in the air. |
| Grow | Both hands, fisted and horizontally-oriented with palms facing upwards, are each moved upwards in front of the shoulders concurrently until reaching eye level as the fingers are released and extended upwards. |
| Hold | Both arms are bent at the elbows so that the forearms extend across the body and are nested in one another with the fingers extended as the body leans to the left. |
| Kick | The index and middle fingers of the right hand, oriented horizontally with the knuckles facing left, are extended downward together. The index finger is then extended leftward independently and brought back to the downward position with the middle finger. |
| Kiss | The right hand, oriented vertically with the palm facing inward and the fingers and thumb pointing upward, is brought vertically to the mouth as the lips are pursed until the fingertips touch it. The hand is then moved outwards away from the mouth and re-oriented horizontally so that the extended fingers point outwards. |
| Knock | The right hand, in a horizontally-oriented fist, is raised above the head to the right of the body and is brought forward in a quick knocking motion three times. |
| Laugh | The right hand is placed on the stomach as the body turns slightly leftward, the mouth is opened into a grin, and the shoulders move up and down three times. |
| Leave | The index and middle fingers of the right hand, horizontally-oriented with the palm facing downward, are loosely extended and moved alternately as the right hand is moved leftward away from the body. |
| Lick | The right hand, in a vertically-oriented fist with the knuckles facing outward, is brought to neck level as the mouth opens and the tongue is extended and moved upwards. |
| Light | The left hand, in a diagonally-oriented fist with the knuckles facing outward, is extended, and the clasped index finger and thumb of the right hand are drawn horizontally across the top of it and then upwards. |
| Listen | The right ear is cupped using right hand as the head is turned leftward so that the right ear faces outward. |
| Look | The right hand, oriented horizontally with palm downward, is placed perpendicular to the forehead as the head is turned to look right and then left. |
| Measure | Both hands in clasp-like grip with thumb and fingertips together are moved apart horizontally. The index finger of the right hand then traces a horizontal line from right to left. |
| Pack | The right and then the left hand, with the palms oriented downward and the fingers curved and extended, are extended to the right and are then concurrently moved to the forward orientation in a horizontal arc as the head and body orient to them. |
| Paint | The right arm, with horizontally-oriented fisted right hand, is moved up and down from above the head to below the waist in a wave-like motion twice. |
| Pay | The left hand with the thumb and fingertips clasped and the palm oriented towards the body is positioned in front of the chest, and the right hand with the thumb and fingertips clasped is moved past it in a rapid downward motion. |
| Plant | The right arm is extended forward as the fingers of the horizontally-oriented right hand, with thumb oriented sideways, are curled under twice. The thumb, index finger, and middle finger are then brought together as the right hand is moved downwards. |
| Play | The left hand, in clasp-like grip with thumb and fingertips together, is positioned above and outward from the left shoulder. The right hand, with fingers loosely extended and palm oriented inward towards body, is moved up and down rapidly several times. |
| Point | The right hand is moved horizontally away from the body as the index finger is extended outward, with the remaining fingers curled into a fist. |
| Pop | The right and then the left hand are raised to head level as the fingers are released and extended with the palms oriented outwards. |
| Pour | Using right arm extended away from the body, the right hand, with fingers extended and curled and palm oriented away from body, is moved in an arc-like motion ending with index finger and thumb oriented downwards. |
| Pray | Both hands, vertically-oriented with palms facing each other and fingers together, are brought towards each other until palms touch while the head is bowed with eyes closed. |
| Propose | The right fist is placed above the left fist, both horizontally-oriented with the palms towards each other and the knuckles facing outward, and the right wrist is rotated upward so that the palm faces outward. |
| Pull | The right hand, in an underhanded grip, is placed beside the left hand, in an overhanded grip, and both hands are concurrently moved leftward. |
| Punch | The right fist is quickly extended forward away from the body and retracted towards the body, and this sequence is repeated with the left fist. |
| Read | Both hands, oriented horizontally with the palms facing each other and the fingers together and extended away from the body, are brought together until the palms touch and are then opened outward so that both palms are facing upward. |
| Rise | The right arm, oriented horizontally with fingers extended together, is lifted upward into a vertical orientation. |
| Roll | The right hand, cupped with palm upward, is shaken back and forth horizontally several times and then extended outward away from the body while releasing the fingers and thumb into an extended position. |
| Run | The arms, with hands fisted, are swung forward beside the moving hips alternately three times. |
| Scan | The right hand, in a fist with the thumb on top, is moved from right to left in front of the left hand, which is oriented vertically with palm facing inward and the fingers extended together. |
| Scratch | The left arm is lifted while extended horizontally in front of the body, and the right hand with fingers curled and extended is brought to the upper left arm and moved along it horizontally several times. |
| Scream | Both hands, with fingers spread apart, are positioned on either side of the face as the eyes and mouth are opened widely. |
| See | Both hands, in clasp-like grip with thumb and fingertips together, are moved concurrently beside the eyes as if putting on glasses. |
| Set | The right arm, vertically-oriented with the palm facing left and the fingers extended upward together, is moved in a downward arc until it is horizontally-oriented, with the palm facing downward and the fingers pointing leftward. |
| Sew | The right hand, in a clasp-like grip with the thumb and fingertips together, is moved in a vertical sine-wave-like motion for three cycles. |
| Shave | The right hand, fisted and oriented vertically, is moved along right jaw from ear to below chin twice. |
| Shoot | The index and middle fingers of the right hand are extended together horizontally away from the body and are abruptly moved up and down twice. |
| Shower | The right hand, with fingers extended and curled and oriented away from the body, is moved in circular scrubbing motions several times near armpit of left arm, which is extended upward vertically. Subsequently, the left hand, with fingers extended and curled and oriented away from the body, is moved in circular scrubbing motions several times near armpit of right arm, which is extended upward vertically. |
| Shrink | Both arms, extended away from the body to either side, with the hands oriented vertically with the palms facing inward and the fingers extended, are moved toward each other horizontally until the hands are about a foot apart. |
| Sing | The right hand, in a vertically-oriented fist, is positioned in front of the chest, and the mouth is opened and the lips moved. |
| Sleep | Both hands, with palms together and fingers extended together, are positioned at a 45° angle as head is tilted rightward until right cheek rests against back of left hand. |
| Smell | Right hand, oriented vertically with palm inward and fingers extended leftward together, is brought in front of face, and fingertips are moved in circles together as head is tilted slightly forward. |
| Smile | Upward-extended index fingers of both hands are brought in front of the center of the mouth and moved apart and upward to trace a smile as the mouth smiles with teeth showing. |
| Smoke | The index and middle fingers, oriented upwards, are brought concurrently to pursed lips and then moved outward as air is expelled from the lips. |
| Squeeze | With the right arm extended outward from the body, the right hand is lifted vertically to shoulder height as the fingers are brought together into a fist. |
| Stir | Right hand, in downward-oriented fist, is positioned above left hand, oriented vertically with palm inward and fingers extended together, and then moved in several horizontal circling motions. |
| Stretch | Both hands, in horizontally-oriented fists with the palms facing outwards, are extended to either side of body in diagonal configuration with left hand above and right hand below as the head is tilted rightward. |
| Study | With both hands horizontally-oriented with the palms facing inward, the right hand is placed in front of the left hand. The right hand is then re-oriented into a vertical orientation and then turned so that the palm faces outwards. The right hand is then brought behind the left and as it is re-oriented into its original horizontal orientation. |
| Subtract | Both hands, with the thumb and fingers loosely pointing downward, are placed beside one another so that the tips of the index fingers are touching. The right hand is then moved rightward away from the left hand. |
| Sweep | Thr right hand, vertically-oriented with the palm oriented away from the body and with the fingers together and the thumb pointing upwards, is moved right and then left in an arc-like horizontal motion. |
| Swim | The right and left hands, with fingers curved and extended together, are alternately brought above the head and forward until they reach chest height, and the fingers are extended forwards. |
| Swing | The right and left hands, fisted and placed end-to-end, are brought behind right shoulder and moved across body with both arms concurrently in horizontal arc towards opposite shoulder. |
| Think | The right hand, with the index finger extended, is moved towards the right side of the head until the index finger touches the right temple twice, and then the right index finger is extended horizontally away from the head as the head is slightly tilted left. |
| Throw | The right hand, cupped, is rapidly brought above the head and forward as the fingers are released and the body turns slightly leftward. |
| Type | Both hands are positioned with palms downward and fingers extended slightly, and fingers are independently moved up and down rapidly. |
| Vote | The right hand with the thumb and index finger clasped is moved in a checkmark-like motion. |
| Wait | The left arm is extended horizontally in front of body with fisted hand, and the index finger of the right hand is tapped against the back of the left wrist three times while looking at it. |
| Walk | The right hand, in a horizontally-oriented fist with the palm downwards and the index and middle fingers extended downwards, is moved away from the body as the index and middle fingers are alternately moved away from the body four times. |
| Wash | The left hand, oriented horizontally with the palm inward and the fingers extended together, is positioned forward and to the left of the body, and the right hand, with fingers curled together touching the thumb, is brought in front of it and moved in circular motions perpendicular to it several times as the body turns slightly leftward. |
| Watch | The right hand is moved towards the face with the index and middle finger extended towards the eyes and is then turned and moved outward with the index and middle fingers extending away from the face. |
| Wave | The right arm is raised with the elbow bent at a 90° angle, and the right hand, with palm facing outward and fingers loosely spread, is moved side to side at the wrist several times. |
| Wink | The left eye is closed and the mouth is opened while smiling as the head is moved slightly rightward. |
| Write | The index finger, middle finger, and thumb of the right hand are extended downward together while clasped and moved in wavy, looping motions above left hand, which is oriented diagonally with the palm facing upwards and the fingers extended together, pointing downwards. |
| Yell | Both hands, curved and oriented vertically with palms facing inward and fingers extended together, are concurrently brought to either side of the mouth, which is opened widely. |
