# Supplementary material for: Semantic Relationships Between Representational Gestures and Their Lexical Affiliates Are Evaluated Similarly for Speech and Text
Source: Front Psychol. 2020 Oct 22;11:575991. doi: 10.3389/fpsyg.2020.575991 (PMC7642993; doi:10.3389/fpsyg.2020.575991)
Supplement: Supplementary file 2 [file Table_2.docx]

**Appendix B**

Semantically-Congruent and Semantically-Incongruent Gesture-Word Pairs

| Semantic congruency | Version | Word | Gesture |
| --- | --- | --- | --- |
| C  IC | 1  2 | Add  Add | Add  Subtract |
| C  IC | 1  2 | Answer  Answer | Answer  Cross |
| C  IC | 2  1 | Ask  Ask | Ask  Stretch |
| C  IC | 1  2 | Bake  Bake | Bake  Sing |
| C  IC | 2  1 | Blow  Blow | Blow  Listen |
| C  IC | 2  1 | Call  Call | Call  Smell |
| C  IC | 2  1 | Catch  Catch | Catch  Kick |
| C  IC | 1  2 | Change  Change | Change  Grade |
| C  IC | 2  1 | Cheer  Cheer | Cheer  Shave |
| C  IC | 2  1 | Choke  Choke | Choke  Kiss |
| C  IC | 1  2 | Clap  Clap | Clap  Propose |
| C  IC | 1  2 | Clean  Clean | Clean  Draw |
| C  IC | 1  2 | Click  Click | Click  Pull |
| C  IC | 2  1 | Climb  Climb | Climb  Run |
| C  IC | 2  1 | Cook  Cook | Cook  Dance |
| C  IC | 1  2 | Cool down  Cool down | Cool down  Dry off |
| C  IC | 2  1 | Cough  Cough | Cough  Pray |
| C  IC | 1  2 | Count  Count | Count  Measure |
| C  IC | 1  2 | Cross  Cross | Cross  Answer |
| C  IC | 2  1 | Cry  Cry | Cry  Yell |
| C  IC | 2  1 | Dance  Dance | Dance  Cook |
| C  IC | 2  1 | Dig  Dig | Dig  Hold |
| C  IC | 1  2 | Dip  Dip | Dip  Plant |
| C  IC | 1  2 | Draw  Draw | Draw  Clean |
| C  IC | 1  2 | Drink  Drink | Drink  Sweep |
| C  IC | 2  1 | Drive  Drive | Drive  Swim |
| C  IC | 1  2 | Dry off  Dry off | Dry off  Cool down |
| C  IC | 1  2 | Eat  Eat | Eat  Write |
| C  IC | 1  2 | Erupt  Erupt | Erupt  Pop |
| C  IC | 1  2 | Flip  Flip | Flip  Light |
| C  IC | 1  2 | Fold  Fold | Fold  Wash |
| C  IC | 2  1 | Go  Go | Go  See |
| C  IC | 1  2 | Grade  Grade | Grade  Change |
| C  IC | 2  1 | Grow  Grow | Grow  Shrink |
| C  IC | 2  1 | Hold  Hold | Hold  Dig |
| C  IC | 2  1 | Kick  Kick | Kick  Catch |
| C  IC | 2  1 | Kiss  Kiss | Kiss  Choke |
| C  IC | 1  2 | Knock  Knock | Knock  Walk |
| C  IC | 1  2 | Laugh  Laugh | Laugh  Wav |
| C  IC | 2  1 | Leave  Leave | Leave  Wink |
| C  IC | 1  2 | Lick  Lick | Lick  Study |
| C  IC | 1  2 | Light  Light | Light  Flip |
| C  IC | 2  1 | Listen  Listen | Listen  Blow |
| C  IC | 2  1 | Look  Look | Look  Swing |
| C  IC | 1  2 | Measure  Measure | Measure  Count |
| C  IC | 1  2 | Pack  Pack | Pack  Shoot |
| C  IC | 1  2 | Paint  Paint | Paint  Punch |
| C  IC | 2  1 | Pay  Pay | Pay  Sew |
| C  IC | 1  2 | Plant  Plant | Plant  Dip |
| C  IC | 2  1 | Play  Play | Play  Throw |
| C  IC | 1  2 | Point  Point | Point  Punch |
| C  IC | 1  2 | Pop  Pop | Popp  Erupt |
| C  IC | 1  2 | Pour  Pour | Pour  Read |
| C  IC | 2  1 | Pray  Pray | Pray  Cough |
| C  IC | 1  2 | Propose  Propose | Propose  Clap |
| C  IC | 1  2 | Pull  Pull | Pull  Click |
| C  IC | 2  1 | Punch  Punch | Punch  Point |
| C  IC | 1  2 | Read  Read | Read  Pour |
| C  IC | 2  1 | Rise  Rise | Rise  Set |
| C  IC | 2  1 | Roll  Roll | Roll  Paint |
| C  IC | 2  1 | Run  Run | Run  Climb |
| C  IC | 1  2 | Scan  Scan | Scan  Stir |
| C  IC | 2  1 | Scratch  Scratch | Scratch  Think |
| C  IC | 2  1 | Scream  Scream | Scream  Smile |
| C  IC | 2  1 | See  See | See  Go |
| C  IC | 2  1 | Set  Set | Set  Rise |
| C  IC | 2  1 | Sew  Sew | Sew  Pay |
| C  IC | 2  1 | Shave  Shave | Shave  Cheer |
| C  IC | 1  2 | Shoot  Shoot | Shoot  Pack |
| C  IC | 1  2 | Shower  Shower | Shower  Sleep |
| C  IC | 2  1 | Shrink  Shrink | Shrink  Grow |
| C  IC | 1  2 | Sing  Sing | Sing  Bake |
| C  IC | 1  2 | Sleep  Sleep | Sleep  Shower |
| C  IC | 2  1 | Smell  Smell | Smell  Call |
| C  IC | 2  1 | Smile  Smile | Smile  Scream |
| C  IC | 1  2 | Smoke  Smoke | Smoke  Squeeze |
| C  IC | 1  2 | Squeeze  Squeeze | Squeeze  Smoke |
| C  IC | 1  2 | Stir  Stir | Stir  Scan |
| C  IC | 2  1 | Stretch  Stretch | Stretch  Ask |
| C  IC | 1  2 | Study  Study | Study  Lick |
| C  IC | 1  2 | Subtract  Subtract | Subtract  Add |
| C  IC | 1  2 | Sweep  Sweep | Sweep  Drink |
| C  IC | 2  1 | Swim  Swim | Swim  Drive |
| C  IC | 2  1 | Swing  Swing | Swing  Look |
| C  IC | 2  1 | Think  Think | Think  Scratch |
| C  IC | 2  1 | Throw  Throw | Throw  Play |
| C  IC | 2  1 | Type  Type | Type  Vote |
| C  IC | 2  1 | Vote  Vote | Vote  Type |
| C  IC | 2  1 | Wait  Wait | Wait  Watch |
| C  IC | 1  2 | Walk  Walk | Walk  Knock |
| C  IC | 1  2 | Wash  Wash | Wash  Fold |
| C  IC | 2  1 | Watch  Watch | Watch  Wait |
| C  IC | 1  2 | Wave  Wave | Wave  Laugh |
| C  IC | 2  1 | Wink  Wink | Wink  Leave |
| C  IC | 1  2 | Write  Write | Writ  Eat |
| C  IC | 2  1 | Yell  Yell | Yell  Cry |
